# Evaluation of a Propolis Water Extract Using a Reliable RP-HPLC Methodology and *In Vitro* and *In Vivo* Efficacy and Safety Characterisation

**DOI:** 10.1155/2013/670451

**Published:** 2013-04-24

**Authors:** Bruno Alves Rocha, Paula Carolina Pires Bueno, Mirela Mara de Oliveira Lima Leite Vaz, Andresa Piacezzi Nascimento, Nathália Ursoli Ferreira, Gabriela de Padua Moreno, Marina Rezende Rodrigues, Ana Rita de Mello Costa-Machado, Edna Aparecida Barizon, Jacqueline Costa Lima Campos, Pollyanna Francielli de Oliveira, Nathália de Oliveira Acésio, Sabrina de Paula Lima Martins, Denise Crispim Tavares, Andresa Aparecida Berretta

**Affiliations:** ^1^Laboratório de Pesquisa, Desenvolvimento e Inovação (P, D & I)-Apis Flora Industrial e Comercial LTDA, Rua Triunfo, 945, 14020-670 Ribeirão Preto, SP, Brazil; ^2^Departamento de Química, Faculdade de Filosofia Ciências e Letras de Ribeirão Preto, Universidade de São Paulo, Avenida Bandeirantes, 3900, 14040-901 Ribeirão Preto, SP, Brazil; ^3^Instituto de Química, Universidade Estadual Paulista, Rua Francisco Degni, 55, 14800-900 Araraquara, SP, Brazil; ^4^Departamento de Ciências Farmacêuticas de Ribeirão Preto, Universidade de São Paulo, Avenida do Café, s/n, 14040-903 Ribeirão Preto, SP, Brazil; ^5^Universidade de Franca, Avenida Dr. Armando Salles de Oliveira, 201-Parque Universitário, 14404-600 Franca, SP, Brazil

## Abstract

Since the beginning of propolis research, several groups have studied its antibacterial, antifungal, and antiviral properties. However, most of these studies have only employed propolis ethanolic extract (PEE) leading to little knowledge about the biological activities of propolis water extract (PWE). Based on this, in a previous study, we demonstrated the anti-inflammatory and immunomodulatory activities of PWE. In order to better understand the equilibrium between effectiveness and toxicity, which is essential for a new medicine, the characteristics of PWE were analyzed. We developed and validated an RP-HPLC method to chemically characterize PWE and PEE and evaluated the *in vitro* antioxidant/antimicrobial activity for both extracts and the safety of PWE via determining genotoxic potential using *in vitro* and *in vivo* mammalian micronucleus assays. We have concluded that the proposed analytical methodology was reliable, and both extracts showed similar chemical composition. The extracts presented antioxidant and antimicrobial effects, while PWE demonstrated higher antioxidant activity and more efficacious for the most of the microorganisms tested than PEE. Finally, PWE was shown to be safe using micronucleus assays.

## 1. Introduction 

Natural products, particularly those of plant origin, are an important source of therapeutic agents. Currently, about 25%–30% of all therapeutic drugs available are derived from natural plant, microbe, or animal compounds. Recent evidence from the pharmaceutical industry shows that for some complex diseases, natural products represent an extremely valuable source for the production of new chemical compounds, since they represent structures selected by evolutionary mechanisms over millions of years [1]. In this context, the products obtained from *Apis mellifera*, like honey, royal jelly, pollen, or propolis, have been widely used since ancient times for their therapeutic properties. Among the various bee products available, propolis represents a great prospect for the pharmaceutical, cosmetic, and food industries.

Working in the field of propolis research, several groups have studied its antibacterial properties [2–4]. Besides its antibacterial activity, propolis demonstrates other pharmacological properties, such as antifungal [5, 6], antiviral [7], antioxidant [8], wound healing [4], and anti-inflammatory activities [9], among others. Most of the studies available in the literature are carried out with propolis ethanolic extract (PEE), and, therefore, little is known about the biological activities of the propolis water extract (PWE). The most common propolis extracting process uses ethanol as solvent [10] including different mixtures of water and ethanol. It is important to point out that propolis water extract obtained from direct extraction of raw material with water resulted in less extractable matter when compared to other solvents, even as flavonoid content and antimicrobial activity [11]. In a previous study, our group demonstrated the anti-inflammatory and immunomodulatory activities of PWE used in the present study [12]. PEE has some disadvantages such as a strong taste and adverse reactions or intolerance to the alcohol [10]. By contrast, PWE has only been characterised in a few reports and has demonstrated to have a higher antioxidant activity than PEE [13].

Considering that propolis is a complex bee derivative product, with a chemical composition closely associated with plant origin, it is difficult to compare results obtained in different studies, due to variation in origin, sazonality, extractions, and the standardization of processing which may interfere with the chemical profile and biological properties of the extract [14]. The chemical composition and botanical origin of Brazilian green propolis has been extensively studied. The chemical profile of green propolis has been linked to a wide range of phenolic compounds, many of them also found in *Baccharis dracunculifolia*, such as cinnamic acid derivatives, phenolics acids, flavonoids, and the prenilated compound artepillin C [15]. Many chromatographic methods are described in the literature, but high-performance liquid chromatography coupled with photodiode array detection (RP-HPLC) currently represents the most widely used technique for the analysis of phenolic compounds in propolis raw material and extracts [15–17]. Nonetheless, there is a lack of information considering validation of methodologies used for the characterization of PWE. As a consequence of the requirement for reliable methods to simultaneously quantify phenolics in both PEE and PWE, the present study demonstrates the validation of a precise and accurate analytical method, using RP-HPLC. Additionally, the similarity between PEE and PWE is demonstrated.

Besides demonstrating the desired effects, the development of new medicines requires the study and evaluation of the toxicology of these new compounds. This equilibrium between effectiveness and toxicity is essential when a new drug is proposed [18]. Considering the potential of PWE, the present work compared the chemical composition of this new extract with the existing alcoholic extract and evaluated the *in vitro* effectiveness while investigating the *in vitro *and *in vivo* genotoxic potential in mammalian cells using a micronucleus assay.

## 2. Materials and Methods

### 2.1. Chemicals, Solvents, and Propolis Extracts

 Caffeic acid, *p*-coumaric acid, and *trans*-cinnamic acid were purchased from Sigma Aldrich (Saint Louis, USA). Gallic acid was acquired from Synth (São Paulo, Brazil). Aromadendrin-4′-methyl ether was isolated, purified, and provided by Professor Jairo Kenupp Bastos from the University of São Paulo [15]. Isosakuranetin was purchased from ChromaDex (Irvine, Canada), and artepillin C was supplied by Wako Chemicals Industries Co (Osaka, Japan). HPLC-grade methanol was supplied by JT Backer (Mexico City, Mexico), and purified water was obtained using a MilliQ Direct Q-5 filter system (Millipore, Bedford, MA, USA). PEE and PWE were obtained from the same batch of raw propolis of *Apis mellifera* (CAS n. 9009-62-5), collected mainly in the “Cerrado” area of Minas Gerais, but also in São Paulo (SP), Rio Grande do Sul (RS), Paraná (PR), and Santa Catarina (SC) states, Brazil, according to blend composition published in the Revista de Propriedade industrial, no. 1778 of 2005 (patent requested). The PEE and PWE extracts were supplied by Apis Flora Co (Ribeirão Preto, SP, Brazil). PEE was obtained by placing raw propolis at −19 to −21°C for 12 h, then grinding it into a fine powder with a blender. Particle size was standardized by filtering through a 42-mesh sieve. PEE was extracted from the powder using hydroalcoholic solution (7 : 3), with dynamic maceration followed by a percolation process and finally by filtration [6]. The PEE obtained represents 11% (w/v) of dry matter. PWE was prepared according to de Andrade et al. [19] with some modifications, and, basically, the propolis raw material was extracted using hydro alcoholic solution (7 : 3), with dynamic maceration followed by percolation and finally by filtration. The PEE carefully obtained is then concentrated in a rotary evaporator under controlled temperature (40–60°C) and reduced pressure. After the complete solvent evaporation (80%–90% of dry matter), the propolis soft extract obtained was subsequently alkaline hydrolyzed and resolubilized in water. The main important consideration for both extracts here, PEE and PWE, is that both were extracted with similar procedure (hydroalcoholic solution), and only PWE was resolubilized in water after hydrolise.

#### 2.1.1. PEE and PWE Yield

 Propolis raw material was evaluated considering quality control parameters according to a methodology published and requested by Brazilian Ministry of Agriculture [20]. So, in this way, several tests were done, considering wax, ash, moisture, mechanical mass, propolis extractable matter (Soxhlet extraction) contents, and others. The relation of propolis extract and propolis raw material was done by dividing the final volume of extract (mL) to raw propolis mass (g) employed. To calculate the yield of PEE and PWE production, it was considered the results of propolis extractable matter obtained by Soxhlet extraction. Considering the results of propolis in raw material and assuming this value like all extractable material present in the raw propolis, it is possible to compare the efficiency of the extraction process and the yield in the extraction methodologies proposed. 

### 2.2. Chromatographic Apparatus and Analytical Conditions

 Instrumentation consisted of a Shimadzu Liquid Chromatograph, LC-20AT quaternary delivery system, equipped with an SIL-20A autosampler, a CTO-10AC column oven, and a DAD-SPD-M20A photodiode array detector (Kyoto, Japan). Analytical conditions were optimized based on previous studies by de Sousa et al. [15] and used a reverse-phase Shim Pack CLC-ODS (C_18_) analytical column (250 mm × 4.6 i.d, and a particle size of 5 *μ*m) from Shimadzu (Tokyo, Japan), protected by a precolumn from the same stationary phase and supplier. The optimized mobile phase consisted of a linear gradient of purified water acidified with 0.1% of formic acid, pH 2.7 (A), and methanol (B), ranging from 20% to 80% of B, within 70 minutes. The flow rate and detection wavelength were 0.8 mL/min and 275 nm, respectively. For all analyses, 10 *μ*L of the sample and gallic acid (100 *μ*g/mL) internal standard were used. Peaks were assigned by comparison with authenticated standards as well as being based upon the retention time and UV spectra, of both the standards and samples, under the same analytical conditions.

### 2.3. Standard Solutions, Calibration Curve, and Sample Preparation

 A standard solution containing all chemical markers was prepared and sequentially diluted to achieve the final concentrations required for the calibration curve as displayed in Table 1. A fixed volume of a gallic acid solution (2 mg/mL) was added as an internal standard to each calibration point, in order to achieve a final concentration of 20 *μ*g/mL. All solutions were injected in triplicate, and the obtained curves were used for sample quantification and to validate the reproducibility of the method. 

A total of 200 mg PEE or PWE was added to 10 mL volumetric flasks containing 5 mL of methanol and 100 *μ*L of the internal standard (a methanolic solution of gallic acid 2 mg/mL). The samples were sonicated for 15 minutes in an ultrasound bath, and the 10 mL volume was completed with purified water acidified with formic acid to pH 2.7. After homogenization, samples were filtered through a 0.45-*μ*m cellulose filter, and 10 *μ*L were injected into the HPLC equipment. 

### 2.4. Method Validation

 The described method was validated according to the International Conference on Harmonization guidelines [21] and conformed to the Brazilian rules for analytical method validation [22]. The parameters evaluated included selectivity, linearity, precision (repeatability and intermediate precision), detection, and quantitation limits.

*Selectivity*. was determined by analyzing the separation and resolution of the main peaks of the propolis samples (PEE or PWE) and standard solutions of all the chemical markers used in this study. The ability of the method to distinguish the analyte among possible interferences was also assessed. 

*Linearity.* was determined by examining the correlation coefficient (*r* ^2^) of the linear regression line for the response versus concentration of the calibration curves prepared as described in Table 1. 

*Detection and Quantitation Limits.* were calculated by determining the signal-to-noise ratio of a low concentration solution containing all the standards used in this study and establishing the minimum concentration at which the analytes can be reliably detected or quantified. In order to estimate the detection limit (LODs) and quantitation limit (LOQs), a signal-to-noise ratio between 3 : 1 for LODs and 10 : 1 for LOQs was used. 

*Precision.* was estimated by evaluating the intraday precision (repeatability) and interday repeatability (intermediate precision) of analyses carried out on two different and consecutive days. For both tests of precision, a set of six replicates at 100% of the test concentration was prepared and analysed for PEE and PWE. Results were expressed in terms of the standard deviation and relative standard deviation (coefficient of variation).

### 2.5. Determination of Total Phenol Content

 The total phenol content of the propolis extracts were estimated using a colorimetric assay based on the procedure described by Waterman and Mole [23] with some modifications. Samples of each extract (0.5 mL) were diluted in volumetric flask of 50 mL with purified water. Aliquots of 1.0 mL were transferred to 10.0 mL of purified water in volumetric flask of 50 mL. These samples were reacted with Folin-Denis reagent and 35% sodium carbonate for 30 minutes at room temperature and protected from the light. Subsequently, absorbance was measured at 760 nm (*n* = 3) using a UV-visible (UVmini-1240 spectrophotometer-Shimadzu, Tokyo, Japan). Total phenol content was calculated via comparison to gallic acid standards, and the results were expressed as milligrams of gallic acid per mL of extract.

### 2.6. Determination of Total Flavonoid Content

 An aluminium chloride colorimetric assay was used to determine total flavonoid content, as described by Funari and Ferro [24]. Samples of each extract (1.0 mL) were diluted in methanol in volumetric flasks of 10.0 mL. Aliquots of 0.4 mL were transferred to 25.0 mL of volumetric flasks with methanol and were reacted with aluminium chloride for 30 minutes at room temperature and protected from the light. The absorbance of the reaction mixture was measured at 425 nm. Total flavonoid content was calculated via comparison with quercetin standards, and the results were expressed in milligrams of quercetin per mL of extract.

### 2.7. Antioxidant Assays

#### 2.7.1. DPPH Radical Scavenging Assay

The stable DPPH radical was utilized to determine ability of the extracts to scavenge free radicals [25]. Solutions of different concentrations of propolis extracts were prepared. Aliquots for each propolis extract were dissolved in methanol and mixed with 1 mL acetate buffer (0.1 M, pH 5.5), 1 mL ethanol, and 1 mL DPPH solution (250 *μ*M). The mixture was mixed vigorously in the dark for 30 minutes. The reduction of the DPPH-radical was determined by measuring the decrease of absorption at 517 nm (UV-visible spectrophotometer). The DPPH scavenging effect was calculated as a percentage of DPPH discoloration using the equation: percentage of scavenging effect = [(ABS_DPPH_ − A_*Ext*⁡_)/ABS_DPPH_] × 100. The extract concentration providing 50% inhibition (IC_50_) was calculated from the graph plotting the scavenging effect (percentage) against extract concentration.

#### 2.7.2. Ferric Reducing Power Assay

 The reducing power of PEE and PWE was measured following the method described by Oyaizu [26]. The different concentrations of the extracts, in a 2.5 mL volume were mixed with 2.5 mL sodium phosphate buffer (0.2 M, pH 6.6) and 2.5 mL of 10 mg/mL potassium ferricyanide. The mixture was incubated at 50°C for 30 minutes. After that, 2.5 mL of 100 mg/mL trichloroacetic acid was added, and the mixture was centrifuged for 10 minutes. Subsequently, 2.5 mL of the upper layer was mixed with 2.5 mL of deionized water and 0.5 mL of 1.0 mg/mL of ferric chloride and the absorbance measured at 700 nm (higher absorbance indicate higher reducing power) in a UV-visible spectrophotometer (UVmini-1240 Shimadzu). Extracts providing IC_50_ were calculated from the graph plotting absorbance against extract concentration.

### 2.8. Antibacterial Assay

To evaluate the antibacterial activity of PEE and PWE, the broth macrodilution method recommended by the Clinical and Laboratory Standards Institute [27] was used, with some modifications. In this study, the following microorganisms were used: *Staphylococcus aureus* ATCC 25923, *Staphylococcus aureus* ATCC 43300, *Staphylococcus epidermidis *ATCC 14990, *Streptococcus pneumoniae* ATCC 49619, *Escherichia coli *ATCC 25922, and *Haemophilus influenzae *ATCC 9006.

Samples were serially diluted in test tubes (13 × 100 mm) with 1 mL of culture medium. After dilutions were made, 1 mL of microbial suspension (10^6^ CFU/mL) was added to each tube. The final inoculum concentration in each test tube was of approximately 5 × 10^5^ CFU/mL. The final dilutions of the samples ranged from 1 : 2 to 1 : 512.

Due to the turbidity of the test broth when PEE or PWE were diluted in the culture medium, it was not possible to determine the minimum inhibitory concentration (MIC). Therefore, the antibacterial activity of the samples was assessed by means of the minimum bactericidal concentration (MBC) which was determined by subculturing 20 *μ*L aliquots from each sample in the broth dilution series onto agar plates. Mueller Hinton agar (Difco, Detroid, MI, USA) was used for the test with *S. aureus*, *S. epidermidis,* and *E. coli*. The plates were incubated at 35°C aerobically for 24 h. Mueller Hinton agar with 5% sheep's blood (Plast Labor, Rio de Janeiro, RJ, Brazil) and *Haemophilus* Test Medium agar (Plast Labor, Rio de Janeiro, RJ, Brazil) were used for the test with *S. pneumoniae* and *H. influenzae*, respectively. The plates were incubated at 35°C in 5% CO_2_ for 24 h. After the incubation period, the MBC was determined. MBC was defined as the lowest concentration of the extract required to kill all the individuals of the microorganism being tested. The MBC values were expressed in *μ*g/mL for total flavonoid content present in the samples in the dilution that propolis was able to kill the microorganisms. Each effective dilution was considered by each flavonoid value of triplicate. The medium followed by standard deviation was calculated for the results expression and statistical analysis (medium ± SD).

### 2.9. *In Vitro* Safety Test System

The V79 cells were grown in monolayer plastic culture flasks (25 cm^2^) in 10 mL HAM-F10 and DMEM (both from Sigma-Aldrich) (1 : 1) culture medium supplemented with 10% fetal bovine serum (Nutricell), antibiotics (0.01 mg/mL streptomycin, and 0.005 mg/mL penicillin, Sigma-Aldrich), and 2.38 mg/mL Hepes (Sigma-Aldrich) at 37°C in refrigerated chamber type BOD (biochemical oxygen demand). Cells from the 4th passage were used for all experiments and were performed in triplicate.

To determine the PWE concentration to be used in the *in vitro *assay, the Colorimetric Toxicology Kit (XTT, Roche Diagnostics) was used. 10^4^ cells were added to the 96 wells of a microtitre plate. Each well received 100 *μ*L of medium HAM-F10/DMEM (1 : 1) containing PWE ranging from 3.12 to 400 *μ*g/mL in concentration. Negative controls (no treatment) and positive (25% dimethyl sulfoxide, DMSO, Sigma-Aldrich) were included. After incubation for 24 h at 37°C, the culture medium was removed and cells were washed with 100 *μ*L phosphate buffered saline (PBS). Subsequently, 100 *μ*L of HAM-F10 medium without phenol red and 25 *μ*L of XTT were added to each well. The microtitre plates were incubated at 37°C for 17 h in the dark. Sample absorbance was determined using a multiplate reader (ELISA-Tecan-SW Magellan 5.03 versus STD 2PC) set at a wavelength of 492–690 nm. Cell viability was expressed as a percentage of viable cells, and the negative control was considered to represent 100%.

The three highest PWE concentrations which did not demonstrate cytotoxicity in the XTT assay (6.25, 12.5, and 25.0 *μ*g/mL) were chosen for the micronucleus test. Negative (no PWE) and positive control (methyl methanosulfonate, MMS, 44 *μ*g/mL) groups [28] were also included. Approximately 500,000 cells were first inoculated into culture flasks and incubated at 37°C for 1 h in B.O.D. The cell cultures were then submitted to the different PWE or control treatments and incubated for 24 h at 37°C in B.O.D. After treatment, the cell cultures were washed with PBS and added to culture flasks containing 5 mL complete culture medium (10% fetal bovine serum) and cytochalasin B (CTB, 3 *μ*g/mL, Sigma-Aldrich). After 17 h incubation, the cells were trypsinized with 0.5 mL of ATV, centrifuged, the supernatant discarded, prior to adding a 1% sodium citrate solution. The cells were then centrifuged, the supernatant discarded and the pellet was then fixed with a solution of methanol and acetic acid (3 : 1) for 24 h. Two to three drops of fixed cell suspension were dropped onto microscope slides. After drying at room temperature, the slides were stained for 5 minutes in 3% Giemsa solution diluted in phosphate buffer pH 6.8. The cells were analysed via light microscopy (Microscopy description required) using a 100x immersion objective. For each culture, 1000 binucleated cells (equaling 3000 cells per treatment) were analysed and the number of cells which contained 0, 1, 2, 3, or more micronuclei counted [29]. The cytotoxicity of the treatment in the micronucleus test was accompanied by the calculation of the rate of nuclear division (NDI). A total of 500 cells with well-preserved cytoplasm were evaluated per culture (equaling 1500 cells per treatment), and the number of cells containing scores of 1–4 nuclei was counted. The NDI was calculated according to [30] using the following formula, where *M*1–*M*4 is the number of cells with 1, 2, 3, and 4 nuclei, respectively; and *N* is the total number of cells counted

(1) $$\begin{array}{c} \text{NDI}=\frac{\left[M1+2\left(M2\right)+3\left(M3\right)+4\left(M4\right)\right]}{N}. \end{array}$$

### 2.10. *In Vivo* Safety Test System

Male Swiss mice weighing approximately 30 g, provided by the animal house of the Faculty of Pharmaceutical Sciences, University of São Paulo, Ribeirão Preto, São Paulo State, Brazil, were used for the experiments. The animals were kept in plastic boxes inside a controlled environment set at 22 ± 2°C with 50 ± 10% humidity and on a 12 h light-dark cycle, with standard rat chow and water being available *ad libitum*. 

The animals were divided into five treatment groups each containing six males, including a positive control group (cyclophosphamide, CPA, Sigma-Aldrich, 50 mg/kg body weight (b.w.)) [31], a negative control group (water), and three groups treated with PWE dissolved in distilled water at doses of 7.0, 14.0, and 21.0 mg/kg b.w. The doses of extract used in this study were selected taking the anti-inflammatory effects of PWE, as proposed by Machado et al. [12], into consideration and were administered to the animals by *gavage *for seven days (0.5 mL/animal/day). The positive control group received distilled water by* gavage*, and on the seventh day of treatment CPA was administered intraperitoneally (0.3 mL/animal). Body weight and water consumption were measured throughout the experimental period. The animals were euthanized 24 h after the last treatment. Micronucleated polychromatic erythrocytes (MNPCEs) were obtained according to the technique described by Mac Gregor [32]. The frequency of MNPCEs was obtained for each sample by analysing 2000 polychromatic erythrocytes (PCEs) per animal via light microscopy. The cytotoxicity of the treatments was assessed by calculating the NDI (PCE/(PCE + NCE) (normochromatic erythrocyte). A total of 400 erythrocytes per animal were analysed.

### 2.11. Statistical Analysis

The analysis of variance (ANOVA–*two way*) and Bonferroni multiple comparison were performed with a level of significance of 5%, including the analysis of the results for MBC of PWE and PEE for each microorganism. Comparison of PEE and PWE according to MBC values (considering all microorganisms), the analysis of phenolics and flavonoids contents were done using unpaired Student *t*-Test, with 95% of significance. The results of *in vitro* and *in vivo* safety were evaluated using Tukey test with a significance level of 5%. Statistical analysis was performed using Prism 4 (Graph Pad).

## 3. Results

### 3.1. Validation of HPLC Methodology

**Figure 1:**
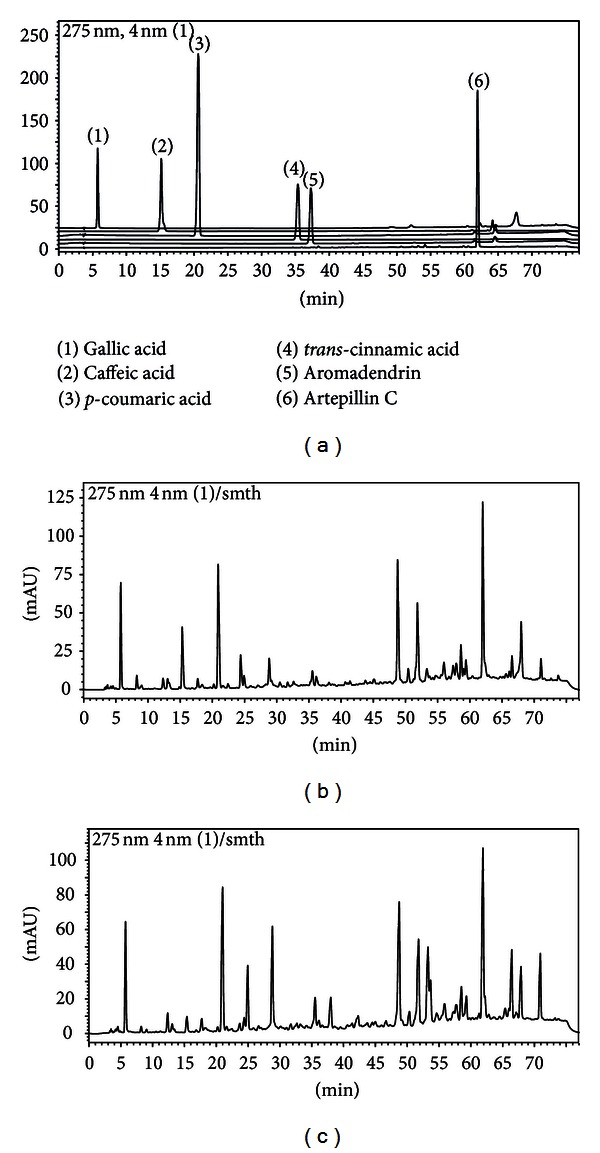
Chromatographic profiles of the five phenolic compounds (a) including the internal standard gallic acid: (1) internal standard; (2) caffeic acid; (3) *p-*coumaric acid; (4) cinnamic acid; (5) aromadendrin, and (6) artepillin C; (b) propolis water extract, and finally, (c) propolis ethanolic extract.

The RP-HPLC methodology proposed showed a good separation of the standards researched in the propolis samples (Figures 1(a)–1(c)). The HPLC fingerprints reveal caffeic acid with 15 min of retention time, *p*-coumaric acid (21-22 min), *trans*-cinnamic acid (36 min), the flavonoid aromadendrin-4′-methyl ether (38-39 min), and finally, artepillin C with 62 min (Figure 1(a)). It is possible to compare Figures 1(b) and 1(c), where it can be seen the fingerprint similarities between PWE and PEE, respectively. The values for linearity, detection and quantitation limits are displayed in Table 2.

The results for inter- and intra-day precision displayed in Table 3 show that the procedure demonstrated good repeatability for multiple samples, including both in PEE and PWE. The relative standard deviations for all chemical markers, for each sample, were lower than 5%, as except for caffeic acid in an aqueous extract (8.25 and 6.44 mg/mL). 

### 3.2. Chemical Profile and Standards Quantification

**Figure 2:**
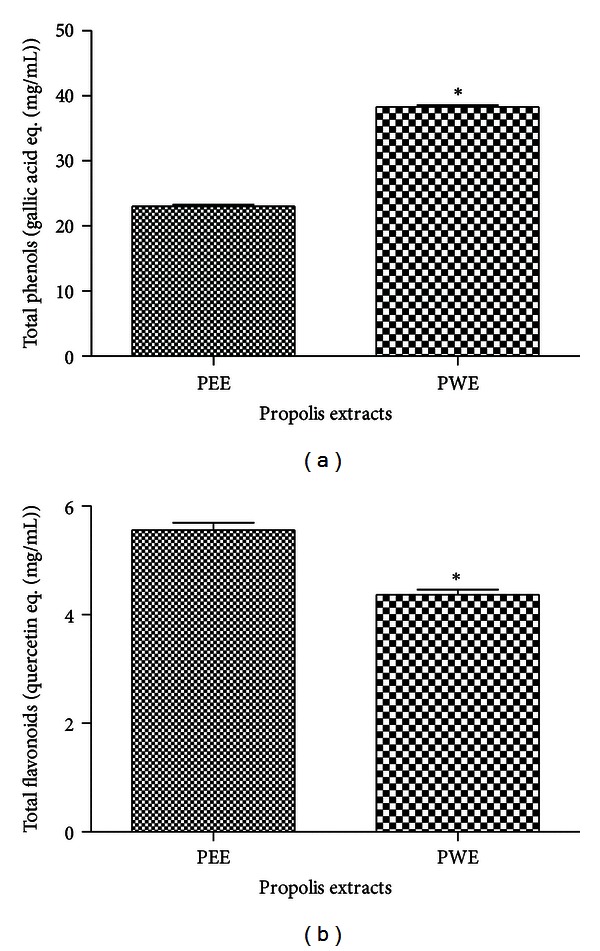
Phenolic and flavonoids contents of PEE and PWE presentation. (a) Total phenolic compounds like gallic acid equivalents (mg/mL) (*n* = 3). (b) Total flavonoids compounds expressed like quercetin equivalents (mg/mL) (*n* = 3). *P < 0.05 (unpaired Student *t-*test, *α* = 0.05).

Propolis raw material used in the present study demonstrated 49.86 ± 2.87%w/w of propolis extractable matter by Soxhlet extraction. PEE and PWE presented 11.0% w/v of propolis dry matter, with propolis: extract relation of 1 : 4. The yield for both was 89.7%, since both were originated from the same extraction process. Considering that PWE possess more steps than PEE, and PWE was obtained from PEE, 100% of yield was obtained in this phase because all PEE was transformed in PWE without loses. Total phenols found for PEE and PWE was, respectively, 23.0 ± 0.25 mg/mL and 38.3 ± 0.25 mg/mL, demonstrating a higher phenolic content in PWE (*P* < 0.05). Flavonoid content for PEE was 5.56 ± 0.197 mg/mL and 4.37 ± 0.091 mg/mL for PWE, fact that supports higher quantities in PEE (*P* < 0.05) (Figure 2). 

### 3.3. Antioxidant Assays

IC_50_ values were very low indicating the high antioxidant activity of the extracts, since our results demonstrated that the IC_50_ value to known food antioxidant BHT (butylated hydroxytoluene) was 4.43 ± 0.03 *μ*g/mL.  Table 4 shows the IC_50_ values for PEE and PWE, demonstrating that both extracts had significant DPPH radical scavenging activity. However, PWE exhibited a higher antioxidant activity than PEE (*P* < 0.001).

The ferric reducing antioxidant ability of an extract or compound may serve as a significant indicator of its potential antioxidant activity. The reducing power was measured by the direct reduction of Fe^+3^(CN^−^)_6_ to Fe^+2^(CN^−^)_6_ and was determined by measuring absorbance resulting from the formation of the Perls Prussian Blue complex following the addition of the excess ferric ions (Fe^+3^). The extract or compound that provided a 50% reduction in absorbance (IC_50_) was calculated from the graph plotting absorbance against the concentration of the extract or compound in the solution. The resulting IC_50_ for PEE and PWE are presented in Table 5. PWE demonstrated higher antioxidant activity than PEE (*P* < 0.001), corroborating the result found using the DPPH method. 

### 3.4. Antibacterial Assay

Gram-positive (*S. aureus* ATCC 25923, *S. aureus* ATCC 43300, *S. epidermidis,* and *S. pneumoniae*) and Gram-negative bacteria (*E. coli* and *H. influenzae*) were used in this study. PEE and PWE presented antibacterial activity against the majority of the microorganisms tested (Table 5). However, PWE was more efficacious than PEE (*P* < 0.001) for the most of microorganisms.* H. influenzae *was the most susceptible microorganism to the extracts (*P* < 0.01), while *E. coli *was the most resistant (*P* < 0.01).

### 3.5. *In Vitro* and *In Vivo* Safety Evaluation of PWE

**Figure 3:**
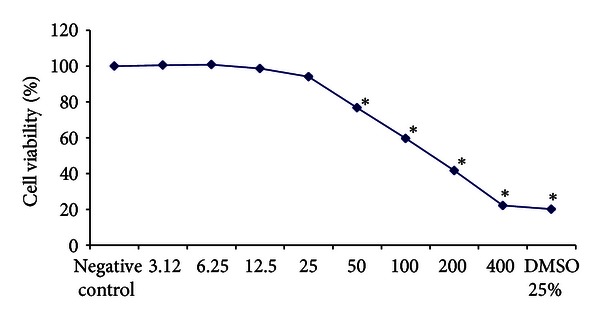
Percentages of viable V79 cells after exposure to different concentrations (*μ*g/mL) of propolis water extract for 24 h, determined by the XTT colorimetric assay. (*) *P* < 0.05.

The percentage of viable V79 cells after treatment with the PWE is shown in Figure 3. The cultures treated with concentrations greater than 25.0 *μ*g/mL of extract showed a cytotoxic effect.

Table 6 shows the frequency of micronuclei in cell cultures and Swiss mice bone marrow samples after the treatment with different concentrations of PWE, and the respective controls. The cultures and animals treated with the propolis extracts showed no difference in micronuclei frequency when compared to the negative control (*P* > 0.05), and therefore present no genotoxicity under these conditions. The NDI values observed in groups treated with the propolis extracts did not show significant differences when compared to negative control group, indicating the absence of cytotoxic treatments under the conditions used (Table 6).

The data obtained for initial and final body weight, weight gained, and water consumption, from the different animals groups, are shown in Table 7. No statistically significant difference was observed for these variables, revealing that the PWE, at the used doses, did not show any signs of toxicity to animals.

## 4. Discussion

Taking into account that some disadvantages are present with alcohol in cosmetic, food, and pharmaceutical preparations, this work aimed to develop a propolis water extract with chemical composition similar to the alcoholic ones, however without alcohol inconvenient. In this work, this objective was reached as it can be observed in the chemical fingerprinting and physical chemical parameters presented. The process used to obtain PEE and PWE produced 89.7% of yield considering propolis extractable matter in raw material, and, because PWE was obtained from PEE, 100% of efficiency was obtained with these further steps. Soxhlet extraction was the more efficacious process when compared to maceration [33]. The comparison of propolis extractable matter from raw propolis obtained in the present case (49.86 ± 2.87% w/w) was lower than the findings of Cunha et al. [33] with 57.65 ± 1.96% w/w of dry matter of raw propolis obtained from Tuiuti, São Paulo State, Brazil. Therefore, maceration is a very common process used to obtain propolis extracts, and the results found corroborate that this is less efficacious than Sohxlet extraction. Other advantageous processes were proposed to produce propolis extracts like ultrasound and microwave extraction [34], and in some cases, the nanofiltration step was used after the maceration extraction [10]. However, thinking about further industrial uses, maceration can be the most simple and affordable technique able to offer PEE and PWE extracts. 

The validated method proposed was based upon a previously published methodology developed by de Sousa et al. [15]. For the HPLC method, several parameters were optimized for the presented study, that is, mobile phase, the gradient method, the internal standard, and the duration of the analysis. These optimizations considered the possible use by other analytical laboratories, keeping in mind economic and environmental costs and operator safety. The first considerable change from the previous method was the substitution of the buffer in the mobile phase to an acidified mobile phase without salt in order to simplify mobile phase preparation and to minimize problems in the equipment such as pump-seal wear or check-valve problems due to salt precipitation, as well as salt precipitation in tubes and columns during subsequently washes and changes in the mobile phase composition [35]. The substitution of acetonitrile by methanol had two main reasons. The first intended to minimize mobile phase eluotropic force to get a more slightly gradient, and the second intended to use a more health and environment friendly solvent, since acetonitrile is more toxic than methanol [36]. Although the time of analysis was increased in 10 minutes, the flow rate could be reduced, allowing the reduction of solvent consume. Finally, due to changes in the mobile phase gradient and composition, the internal standard had to be substituted. So, it was chosen, the gallic acid, because it is easily found in the market and because it has desirable hydrophilic properties, allowing an earlier elution before all the other desired compounds, in a retention time where there is no peaks coeluting. For sample preparation, the procedure is completely different since in this paper we describe the analysis of liquid extracts, and not propolis raw material. For that, it was carefully planned how the sample should be prepared in few and simple steps considering the amount and dilution, as well as its adequacy with the calibration curve range, including the choice of the new internal standard and allowing a reliable quantification. So, the optimized method was applicable for aqueous and hidroalcoholic extracts and it showed good sensitivity, since all chemical markers could be detected and quantified even in the complex propolis extracts.

The chemical composition of propolis water extracts is known to be very different from alcoholic extracts due to the differences in the extraction process [10, 11]. Flavonoids are a large group of phenolic compounds that are more readily extracted with solvents like alcoholic solutions with 70°GL until 96°GL (%v) or methanol [10, 11]. According to HPLC fingerprint, similar results were obtained by Mello et al. [10] and Park and Ikegaki [11], and both observed that with more polar solvents (100% water, e.g.,), propolis extract showed the presence of caffeic and *p*-coumaric acids, while less polar solvents presented substances more lipophilics (compounds obtained after 20 minutes of chromatographic running). With these data ethanolic solutions like extractable solvent are more advantageous when compared with water [10]. However, the HPLC analysis in the presented study showed that PWE possessed all the standard compounds commonly found in PEE, including relatively more apolar compounds such as artepillin C, although quantitative concentrations differed (*P* < 0.05). In this investigation, the two extracts evaluated originated from the same batch of Brazilian green propolis, the same extraction process, however PWE was obtained after hydrolyse of propolis extractable matter of PEE, followed by water solubilisation. Satisfactory results concerning the chemical profile of the propolis water extracts, that is, the presence of the same standards commonly present in alcoholic extracts, were obtained because the extraction origin was the same, fact not observed when water is used as solvent in the extraction of propolis raw material (data not shown). The results obtained (not shown) are in accordance with Mello et al. [10] and Park and Ikegaki [11].

Phenolics compounds are commonly found in both edible and nonedible plants and have been reported to have multiple biological effects, including antioxidant activity [37, 38]. Propolis contains a wide variety of compounds, mainly flavonoids. Flavonoids and other phenolics compounds have been suggested to play a preventive role in the development of cancer and heart diseases [37, 38]. The biological activities of Brazilian propolis have been suggested to be due to the high phenolic acids content [39] while flavonoids are considered to be responsible for the activity of European propolis extracts [40]. The higher antioxidant activity of PWE compared to PEE, as demonstrated in the presented study, was probably due to the higher polyphenol content and the better solubility of the phenol constituents in water, specially the known antioxidant compounds caffeic and *p*-coumaric acids [41], results corroborated by Nagai et al. [42]. However, phenolics in PWE (348 *μ*g/mg) were greater than those obtained by Nagai et al. (168 *μ*g/mg) [42]. 

Brazilian propolis extracts have been shown to possess antibacterial activity, predominantly against Gram-positive bacteria [2–5]. Some Gram-negative bacteria are also susceptible to Brazilian propolis [2], while others are not [3]. In the presented study, *H. influenzae*, which is Gram-negative, was shown to be the most susceptible to both PWE and PEE of the microorganisms evaluated. Therefore, both propolis extracts may be used to supplement the treatment of the diseases caused by* H. influenzae*, such as sinusitis, otitis media, and meningitis. Another Gram-negative species, *E. coli *was the most resistant microorganism to both propolis extracts, which is in agreement with the study by Jorge et al. [3], where the ethanolic extracts of Brazilian green propolis showed no antibacterial activity against *E. coli.*

The presented study also demonstrated the antibacterial activity of PWE and PEE against the causal agents of several common diseases. *S. pneumoniae* is a common cause of pneumonia, sinusitis, otitis media, meningitis, and septicaemia [43]. Both PEE and PWE presented antibacterial activity against *S. pneumoniae*; however, PEE was more efficacious. The extracts also presented antibacterial activity against the staphylococci, including *S. aureus *ATCC 43300, a methicillin-resistant *S. aureus *(MRSA). These strains are resistant to all penicillins, cephalosporins, and carbapenems; that is, they exhibit multidrug resistance. Therefore, they are responsible for several difficult-to-treat infections in humans. The current prevalence of antibiotic-resistant bacteria, such as MRSA, has led to a reevaluation of the use of natural therapeutic products, which may represent an alternative treatment of such disease.

Toxicological genetic tests are essential to evaluate potential risk of compounds within drugs that could be carcinogenic or cause hereditary mutations. It is clear that no single test is capable of detecting all relevant genotoxic agents. Therefore, the usual approach is to carry out a variety of *in vitro* and *in vivo* tests for genotoxicity. Such tests are complementary rather than representing different levels of hierarchy [44]. The micronucleus assay is widely used for the screening of genotoxic potential and allows the detection of both the clastogens and aneugens. 

The results obtained in this study showed that the cell cultures and the animals treated with PWE presented no genotoxic effect in the micronucleus assay. These results are consistent with the studies carried out with green propolis hydroalcoholic extract using chromosomal aberrations assay in Chinese ovary hamster cells (CHO cells) [45] and Wistar rat bone marrow cells [46]. In addition, the topical formulations of standard green propolis extract evaluated by CHO cells chromosomal aberrations assay and Wistar rat peripheral blood micronucleus test showed no mutagenic effect in either test systems [47]. However, Pereira et al. [48] observed genotoxic activity of ethanol extract of propolis when using doses higher than 1000 mg/kg p.c. on Swiss mice peripheral blood using the comet and micronucleus assays. 

## 5. Conclusions

The results of this study demonstrated the development and validation of an HPLC method, comprising five standards, for evaluating propolis water and ethanolic extract composition. Furthermore, we demonstrated that both propolis extracts contained all of the compounds researched, a fact that is very difficult to observe in the literature and it is probably due to the extraction process used. Both extracts showed antioxidant and antimicrobial activities, while PWE demonstrated the most potent antioxidant activity and efficacious for most of the microorganisms tested. Finally, the PWE showed to be safe using *in vitro* and *in vivo* micronucleus assays.

## Figures and Tables

**Table 1:** Concentration range for the calibration curves of the five phenolic compounds used in this study.

Chemical marker	Concentration range in *μ*g/mL							
Caffeic acid	1.06	2.12	4.24	6.36	8.48	10.60	12.72	16.96
*p*-coumaric acid	5.04	10.08	20.16	30.24	40.32	50.40	60.48	80.64
*trans*-cinnamic acid	0.40	0.80	1.60	2.40	3.20	4.00	4.80	6.40
Aromadendrin	2.00	4.00	8.00	12.00	16.00	20.00	24.00	32.00
Artepillin C	10.06	20.12	40.24	60.36	80.48	100.60	120.72	160.96

**Table 2:** Results of linearity, quantitation, and detection limits determined for the five phenolic compounds used in this study.

Chemical marker	Regression curve	*r*	LOD *μ*g/mL	LOQ *μ*g/mL
Caffeic acid	*y* = 0.0417*x* − 0.0136	0.9991	0.39	1.51
*p*-coumaric acid	*y* = 0.0752*x* − 0.0891	0.9993	0.21	0.81
*trans*-cinnamic acid	*y* = 0.1547*x* − 0.0148	0.9993	0.10	0.40
Aromadendrin	*y* = 0.0322*x* − 0.0154	0.9992	0.52	2.00
Artepillin C	*y* = 0.0221*x* − 0.0562	0.9992	0.50	1.93

**Table 3:** Precision results for propolis water extract (PWE) and propolis ethanolic extract (PEE).

Sample	Chemical marker	Intraday repeatability (*n* = 6)			Interday repeatability (*n* = 6)			*F* test
		Average quantity (mg/mL)	RSD	VAR	Average quantity (mg/mL)	RSD	VAR	
	Caffeic acid	0.342	8.25	0.00080	0.362	6.44	0.00054	0.68
	*p*-coumaric acid	1.078	0.44	0.00002	1.075	1.00	0.00012	5.08
PWE	*trans*-cinnamic acid	0.078	2.18	0.00000	0.077	0.61	0.00000	0.50
	Aromadendrin	0.097	3.38	0.00001	0.097	2.48	0.00001	1.88
	Artepillin C	4.393	1.98	0.00758	5.450	1.43	0.00603	1.26

	Caffeic acid	0.267	1.48	0.00002	0.276	0.36	0.00000	0.06
	*p*-coumaric acid	1.418	0.72	0.00010	1.433	0.46	0.00004	0.42
PEE	*trans*-cinnamic acid	0.160	2.84	0.00002	0.152	0.91	0.00000	0.16
	Aromadendrin	0.772	0.97	0.00006	0.810	1.38	0.00013	0.45
	Artepillin C	5.480	3.01	0.02727	5.135	1.77	0.00828	3.29

RSD: relative standard deviation.

VAR: variance.

**Table 4:** The concentration of propolis water (PWE) or ethanolic (PEE) extracts, from Brazilian green propolis, required for a 50% reduction of DPPH or Fe^3+^, representing free radical scavenging and ferric reducing activity, respectively.

Extract	DPPH^•^ scavening		Fe^3+^-Fe^2+^ Reducing	
	IC_50_	*r* ^2^	IC_50_	*r* ^2^
PEE	56.71 ± 2.31	0.9964	282 ± 9.4	0.9971
PWE	33.36 ± 2.22	0.9988	270 ± 3.27	0.9925

Values are mean ± SD obtained from analyses in triplicate.

The values were expressed as *μ*g/mL. Lower IC_50_ values indicate higher antioxidant activity.

**Table 5:** Minimum bactericidal concentration (MBC) of propolis water extract (PWE) and propolis ethanolic extract (PEE) from Brazilian green propolis.

Microorganisms	MBC^a^ (*μ*g/mL)	
	PEE	PWE
*Staphylococcus aureus* ATCC 25923	346.25 ± 0.012*	136.67 ± 0.003
*Staphylococcus aureus* ATCC 43300	173.13 ± 0.006*	68.33 ± 0.002
*Staphylococcus epidermidis* ATCC 14990	346.25 ± 0.012*	136.67 ± 0.003
*Streptococcus pneumoniae* ATCC 49619	43.28 ± 0.002	136.67 ± 0.003*
*Escherichia coli* ATCC 25922	692.5 ± 0.024	1093.3 ± 0.023*
*Haemophilus influenzae* ATCC 9006	43.28 ± 0.002*	17.03 ± 0.000

^a^MBC values (*μ*g/mL) expressed like total flavonoid content. *Significant difference between PEE and PWE (*P* < 0.001).

Values are presented as the mean ± SD (*n* = 3).

**Table 6:** Frequency of nuclear division (NDI), micronucleated binucleated cells (MNCBNs), and micronucleated polychromatic erythrocytes (MNPCEs) after treatment with different concentrations of propolis water extract and their respective controls.

*In vitro* test system		
Treatments (*μ*g/mL)	NDI^a^	MNCBNs^b^
Negative control	1.75 ± 0.05	6.33 ± 0.57
6.25	1.80 ± 0.03	4.33 ± 1.52
12.5	1.76 ± 0.06	4.34 ± 0.57
25.0	1.80 ± 0.02	7.66 ± 0.57
MMS	1.75 ± 0.06	57.60 ± 11.9*

*In vivo* test system		
Treatments (mg/kg b.w.)	NDI^c^	MNPCEs^d^

Negative control	0.68 ± 0.01	3.0 ± 0.0
7	0.65 ± 0.04	4.0 ± 2.0
14	0.68 ± 0.03	4.6 ± 2.0
21	0.60 ± 0.04	4.8 ± 1.6
CPA	0.61 ± 0.02	50.6 ± 7.0*

MMS: methyl methanesulfonate (44 *μ*g/mL); CPA: cyclophosphamide (50 mg/kg b.w.). ^a^500 binucleated cells were analysed per culture, corresponding to a total of 1500 cells per treatment. ^b^1000 binucleated cells were counted per culture, corresponding to a total of 3000 cells per treatment. ^c^400 erythrocytes were analysed per animal, corresponding to a total of 2400 cells per treatment. ^d^2000 erythrocytes were analysed per animal, corresponding to a total of 12,000 cells per treatment.

The values are presented as the mean ± standard deviation.

*Significantly different from the negative control group (*P* < 0.05).

**Table 7:** Mean values for the initial and final weight, weight gained, and water consumption obtained from Swiss mice treated with propolis water extract for 7 days and respective controls. The values are presented as the mean ± standard deviation.

Treatments (mg/kg b.w.)	Initial weight (g)	Final weight (g)	Weight gain (g)	Water consumption (mL/animal/day)
Negative control	21.25 ± 3.18	23.00 ± 3.53	1.75 ± 0.35	4.64 ± 3.66
7	30.08 ± 0.49	30.75 ± 0.82	0.67 ± 0.52	4.17 ± 2.26
14	23.58 ± 1.88	28.67 ± 1.12	5.08 ± 2.15	9.59 ± 3.53
21	28.25 ± 1.72	29.50 ± 2.37	1.25 ± 1.08	9.97 ± 4.26
CPA	25.25 ± 2.81	27.33 ± 2.25	2.08 ± 0.74	3.81 ± 2.72

CPA: cyclophosphamide (50 mg/kg b.w.).
